# Myelination selectively modulates BOLD signal in white matter

**DOI:** 10.21203/rs.3.rs-6597153/v1

**Published:** 2025-05-07

**Authors:** Yurui Gao, Lyuan Xu, Kurt G. Schilling, Soyoung Choi, Ran Chen, Yikang Li, Muwei Li, Zhongliang Zu, Zhaohua Ding, Adam W. Anderson, John C. Gore

**Affiliations:** 1 Vanderbilt University Institute of Imaging Science, Vanderbilt University Medical Center, Nashville, TN, USA, 37232; 2 Department of Biomedical Engineering, Vanderbilt University, Nashville, TN, USA, 37235; 3 Department of Electrical and Computer Engineering, Vanderbilt University, Nashville, TN, USA, 37235; 4 Department of Radiology and Radiological Sciences, Vanderbilt University Medical Center, Nashville, TN, USA, 37232; 5 School of Medicine, Meharry Medical College, Nashville, TN, USA, 37208

**Keywords:** white matter, BOLD, fMRI, fALFF, biophysical model, myelin, hemodynamic, metabolism, neurite, mitochondria

## Abstract

There is increasing recognition that blood oxygenation level dependent (BOLD) signals are detectable in white matter (WM) and reflect an important, heretofore overlooked functional activity in the brain, but their biophysical origins remain understudied and poorly understood. By integrating several disparate, multimodal data sets, we established the associations of resting state BOLD signals with key microstructural, hemodynamic and metabolic features in WM. In particular, we identified the roles of myelination and fiber type in modulating BOLD effects, and derived relationships between measurements of BOLD signal power and cerebral blood volume, flow, oxygen extraction and metabolic rate of oxygen consumption, which are predicted using a simple theory and then verified empirically. Our findings demonstrate that myelin selectively influences the fractional amplitude of low-frequency fluctuations (fALFF) in BOLD signals, and that differences in myelin content account for variations in their temporal spectra and hemodynamic response functions, but these in turn are qualitatively different in association versus projection fibers. Other determinants of BOLD in WM are further revealed by converging biological, genomic and neurochemical evidence, including measurements of neurite and mitochondrial densities. Moreover, analyses of images of the optic nerve from human subjects confirm that BOLD activations evoked by visual stimuli are preferentially localized to unmyelinated portions, with minimal responses in fully myelinated regions of the same nerve, suggesting a myelin-dependent requirement for BOLD effects in WM.

## Introduction

Although they have been largely overlooked for many years, blood oxygenation level dependent (BOLD) signals can be robustly detected in white matter (WM) using functional magnetic resonance imaging (fMRI) methods^1^, and it is clear they vary in response to changes in neural activity^2–5^, are altered in various pathological conditions^6–8^, and so should no longer be treated as nuisance regressors in fMRI analyses^9,10^. Emerging evidence demonstrates that WM BOLD signals represent potentially important and heretofore overlooked indicators of neural activities that are intimately related to how cortical regions communicate, and so should be incorporated into more complete models of brain functional organization.^2,9–11^. However, interpretations of BOLD effects in WM remain unclear, and the biophysical origins of BOLD signals in WM have been little studied. This report attempts to bridge this knowledge gap and provide new insights for interpreting BOLD signals in WM.

BOLD effects in general reflect changes in the tissue concentration of intravascular deoxygenated hemoglobin ([dHb]), which acts as a susceptibility contrast agent and affects the effective transverse relaxation rate $\left(R_{2}^{*}\right)$^12,13^ of tissue magnetization. In WM, changes in [dHb] arise in a manner similar to gray matter (GM) and other tissues and are caused by hemodynamic responses (including changes in cerebral blood flow) to changing demands for energy substrates, particularly oxygen. This is the mechanism by which neural activity in GM modulates the level of dHb, and thus $R_{2}^{*}$, in the cortex, and similar variations in energy demand may produce corresponding changes in WM. If neural activity and oxygen demand suddenly increase, the local [dHb] may transiently increase until incoming arterial blood re-establishes a higher oxygenation level, so the BOLD signal response (the hemodynamic response function or HRF) may be bi-phasic, and a decrease in [dHb] may be delayed by the time required for arterial flow to increase. This model is supported by consistent patterns of task-induced BOLD responses observed in both WM and GM^14^ and the measured MRI signal responses to transient (e.g. event-related) stimuli^15^. At rest, BOLD fluctuations in both tissues show similar power spectra dominated by a specific range of low-frequency components^16^. We thus posit that BOLD signals in WM reflect variations in local hemodynamic responses and metabolism to time-varying energy demands, just as for GM.

The hemodynamic response function (HRF) that describes the signal changes after a transient stimulus in WM is qualitatively similar but differ in detail from that in GM, and exhibits a reduced magnitude, delayed onset, and a more pronounced initial negative dip^15,16^. At rest, the area under this dip inversely correlates with neurite density, a behavior quite opposite from GM^16^, suggesting that the energetic drivers of WM BOLD signals may arise from different tissue components than those in GM. In WM, energy is predominantly used to support both axonal and glial functions, and the fraction of tissue occupied by glial cells is much greater in WM than in GM. Oligodendrocytes, which produce axonal myelin, allocate a significant portion of total energy use to maintain glial membrane potentials^17^. Astrocytes are involved in various activities including synaptic modulation, glycogen metabolism, nutrient supply (e.g. lactate), and ion regulation (e.g., K^+^) following neural activity^17,18^—all of which consume energy and oxygen. Optogenetic studies have shown that astrocytes alone can induce BOLD responses with oxygen consumption^19^. Together, glial cells form a panglial network linking capillaries to myelinated axons, allowing transport of ions and metabolites^20^. These findings suggest that glial-driven metabolic signaling may contribute to WM BOLD effects. These findings also motivate the studies reported below which address the hypothesis that variations in tissue composition and microstructure modulate BOLD signals in WM, determining their relations with hemodynamic and metabolic features.

Multiple research imaging and other types of brain studies, performed across different institutions on large, controlled cohorts of subjects, are publicly available for data analysis. Here, we integrate multi-modality brain atlases obtained from different sources to derive a new understanding of the hemodynamic and metabolic correlates of WM BOLD signals, and the role of WM myelination in modulating these relationships. Specifically, we quantify: (1) relationships between WM resting state BOLD fluctuations in individual WM fiber bundles and measures of cerebral blood volume and blood flow, oxygen extraction fraction and metabolic rate of oxygen consumption, by integrating multimodal neuroimaging data and simple theory; (2) the influence of myelin content and its moderating effect on these relationships, with attention to apparent differences between association and projection fibers, by integrating separate atlases of myelin water and diffusion and functional data; (3) the dependences of BOLD signals, their power spectra and the HRF, on myelin, neurite and mitocohondrial densities; and (4) differences in evoked BOLD signals between unmyelinated and fully myelinated fiber segments produced by a task. The results below provide a compelling interpretation of BOLD metrics in terms of basic metabolic and microstructural characteristics of WM that are inhomogeneously distributed across the brain.

## Results

### 1. Association and projection GM-WM-GM units

**Fig. 1:**
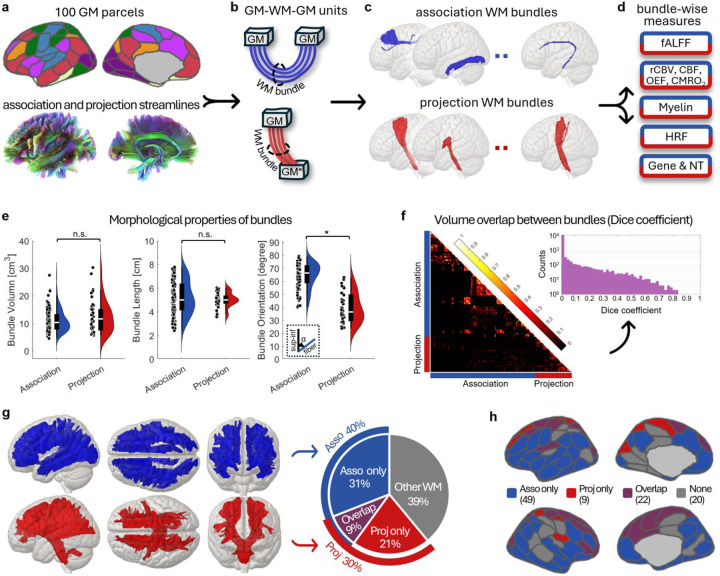
Flowchart for defining association and projection WM bundles and their morphological properties. **a**, GM parcels and WM streamlines from Schafer 100 atlas and HCP1065 atlas used to define WM bundles (see Methods). **b**, Schematic GM-WM-GM motifs representing association (top) and projection (bottom) units; GM* denotes the cerebellar cortex or the cervical portion of spinal cord). **c**, Resulting association and projection WM bundles. **d**, Bundle-wise measures extracted for downstream analyses. **e**, Morphological properties of two types of bundles, including volume, length and orientation (angle deviation from the superior-inferior direction). **f**, Volume overlap between bundles evaluated by Dice coefficients. **g**, Collective volumes of 120 association bundles (blue) and 40 projection bundles (red), and their fractions of total WM volume. **h**, Distribution of GM parcels connected by the 160 WM bundles, showing parcels connected exclusively by association bundles (blue), projection bundles (red), both types (purple), or neither (gray).

To segment and quantify WM BOLD signals we first defined 160 WM bundles by considering how each was connected to GM regions. We defined each GM-WM-GM unit as a structural triplet comprising two GM parcels and a WM bundle connecting them (Fig. 1b). To construct the units, we extracted over 260,000 streamlines from association and projection fibers in the HCP1065 tractography atlas^21^ and reassigned each streamline to a specific bundle according to its GM end-parcels, as defined by the Schaefer 100 parcellation^22^ and supplemented with cerebellar and cervical spinal cord regions (Fig. 1a, b). Following exclusion of bundles containing fewer than 500 voxels (voxel size = 2×2×2 mm^3^), a total of 120 association bundles and 40 projection bundles (Fig. 1c) were retained for downstream analyses (Fig. 1d).

Morphologically, these two types of bundles have no significant differences in volume or length, yet projection bundles were more closely aligned with the superior-inferior direction in MNI space compared to association bundles (Fig. 1e). Bundles could overlap to some extent (Fig. 1f) due to the complexity of WM intrinsic structure. Collectively, all 160 bundles accounted for 61% of total WM volume (Fig. 1g) and were connected to 82 out of 100 GM parcels (Fig. 1h).

### 2. Hemodynamic and metabolic correlates of baseline fALFF in WM

**Fig. 2:**
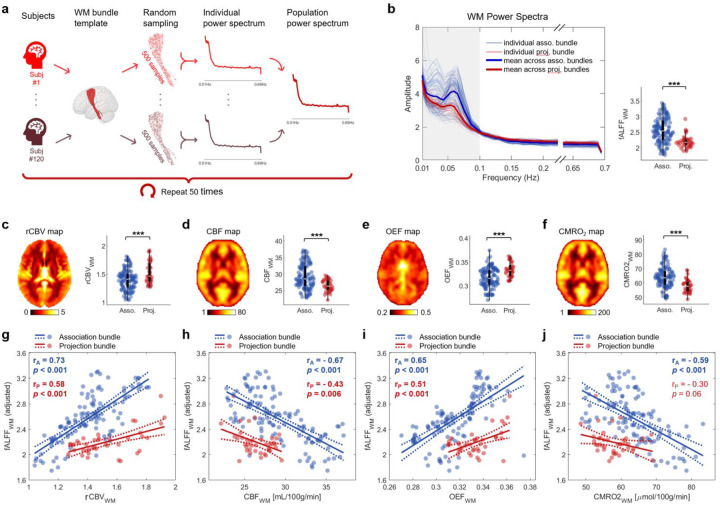
Relationships of bundle-wise fALFF with hemodynamic and metabolic properties in association and projection bundles. **a**, Overview of bundle-wise fALFF computation. **b**, Left: Power spectra of 120 association bundles (blue) and 40 projection bundles (red). Right: Group comparison of WM fALFF between the two bundle types. **c-f**, Population-level maps (left) and group comparisons (right) of rCBV (**c**), CBF (**d**), OEF (**e**) and ${\text{CMRO}}_{2}$ (**f**). *** p<0.001. **g-j**, Scatter plots showing correlations between fALFF and rCBV (**g**), CBF (**h**), OEF (**i**) and ${\text{CMRO}}_{2}$ (**j**), in association (blue) and projection bundles (red).

To identify biophysical factors that affect resting-state BOLD fluctuations in WM, we measured the correlations between the fractional amplitude of low-frequency fluctuations (fALFF^23^) and key hemodynamic and metabolic features. The fALFF, defined as ratio of the average power amplitude in the 0.01 Hz - 0.1 Hz band to the amplitude above 0.1Hz, served as a metric of BOLD fluctuations at rest. We measured the bundle-wise WM fALFF across 120 subjects from the HCP Young Adult database using an unbiased sampling strategy (Fig. 2a and **Fig. S1**; see Methods). Bundle-wise hemodynamic and metabolic features were derived from population-level atlases derived by del Mar Álvarez-Torres *et al*.^24^ and Driver *et al*.^25^: relative cerebral blood volume (rCBV) was obtained by averaging 134 subjects imaged by dynamic susceptibility contrast T2* imaging^24^ (Fig. 2c), cerebral blood flow (CBF), oxygen extraction fraction (OEF), and cerebral metabolic rate of oxygen (CMRO₂) were obtained by averaging 33 subjects assessed using calibrated fMRI^25^ (Fig. 2d–f).

After accounting for bundle volume (**Fig. S2**), we observed a strong positive dependence of fALFF on rCBV in both association bundles and projection bundles (${\text{r}}_{\text{A}}=0.73,p<0.001;{\text{r}}_{\text{P}}=0.58,p<0.001$; Fig. 2g) and on OEF (${\text{r}}_{\text{A}}=0.65,p<0.001;{\text{r}}_{\text{P}}=0.51,p<0.001$; Fig. 2i). Conversely, fALFF was negatively associated with CBF (${\text{r}}_{\text{A}}=-0.67,p<0.001;{\text{r}}_{\text{P}}=-0.43,p=0.006$; Fig. 2h) and ${\text{CMRO}}_{2}$ (${\text{r}}_{\text{A}}=-0.59,p<0.001;{\text{r}}_{\text{P}}=-0.30,p=0.06;$ Fig. 2j). We also observed a significant positive correlation between fALFF and mean transit time (MTT) (**Fig. S3**). Collectively, these findings suggest that greater BOLD fluctuations at rest are associated with lower rates of perfusion, longer time to enter and depart tissue, higher oxygen extraction, and lower oxygen consumption. Moreover, these relations are quantitatively different in different bundle types.

### 3. A biophysical model for interpreting fALFF-metabolic associations

To interpret these empirical observations shown in Fig. 2g–j, we established a biophysical model linking BOLD signal fluctuations to hemodynamic and metabolic dynamics. This model assumes that BOLD fluctuations reflect changes in local [dHb] in tissue, which are induced by variations in metabolic demand associated with neural activity. Suppose $\delta R_{2}^{*}(t)$ is the contribution to the transverse relaxation rate produced by deoxygenated blood at time t. If the BOLD signal is S(t) with a mean value $\overset{‾}{S}$, then for a small change in relaxation rate ($\delta R_{2}^{*}\cdot TE\ll 1$):

(1) $$\delta R_{2}^{*}(t)\approx \frac{1}{TE}\frac{S(t)-\overset{‾}{S}}{\overset{‾}{S}}$$

where TE is the echo time. The fALFF is directly related to the variance of the signal fluctuations within a specific frequency range and thus is related to the variance in $\delta R_{2}^{*}(t)$, denoted as $\sigma_{\delta R_{2}^{*}}^{2}$.

From the perspective of oxygen supply, OEF reflects the fraction of arterial oxygen removed from the blood, so it also determines the fraction of residual hemoglobin in tissue that is deoxygenated. To first order (and also approximately true when oxygenation effects are considered more fully), we have

(2) $$\delta R_{2}^{*}(t)=\beta \cdot OEF(t)\cdot CBV(t),$$

where β is a constant coefficient. Thus, the variance in $\delta R_{2}^{*}(t)$ can be expressed as

(3) $$\sigma_{\delta R_{2}^{*}}^{2}=\beta^{2}\left(CBV^{2}\sigma_{OEF}^{2}+OEF^{2}\sigma_{CBV}^{2}\right),$$

and so will increase with higher values of CBV or OEF, as found empirically.

From a metabolic standpoint, changes in oxygen consumption drive changes in the BOLD signal. $CMRO_{2}$ describes the rate of oxygen use and is related to OEF and CBF by the relationship $CMRO_{2}=\alpha \cdot OEF\cdot CBF$, where CBF is itself related to CBV as CBF=CBV/MTT. Substituting these relations into Eq. (2) yields:

(4) $$\delta R_{2}^{*}(t)=\frac{\beta }{\alpha }CMRO_{2}(t)\cdot MTT(t)$$

$\delta R_{2}^{*}(t)$ (and the signal variation S(t)) is thus a direct measure of the rate of oxygen use at rest. We posit that changes in baseline neural activity cause changes in ${\text{CMRO}}_{2}(t)$ measured by the variance $\sigma_{CMRO_{2}}^{2}$. The variance in $\delta R_{2}^{*}(t)$ is given as

(5) $$\sigma_{\delta R_{2}^{*}}^{2}=\frac{\beta^{2}}{\alpha^{2}}\left(MTT^{2}\sigma_{CMRO_{2}}^{2}+CMRO_{2}^{2}\sigma_{MTT}^{2}\right).$$

In a resting state, if flow fluctuations are primarily due to CBV variations caused by vasodilation or vasoconstriction (e.g., via pericytes) then the variance in $MTT,\sigma_{MTT}^{2}$, is relatively small, and MTT remains nearly constant. Then Eq. (5) simplifies to:

(6) $$\sigma_{\delta R_{2}^{*}}^{2}\approx \frac{\beta^{2}}{\alpha^{2}}MTT^{2}\sigma_{CMRO_{2}}^{2}\;\text{or}\;\frac{\sigma_{\delta R_{2}^{*}}^{2}}{\delta R_{2}^{*2}}=\frac{\sigma_{CMRO_{2}}^{2}}{CMRO_{2}^{2}}$$

This predicts an inverse relationship between fALFF and $CMRO_{2}$ (and CBF), consistent with our empirical findings. Note the assumption that the second term of Eq. (5) is relatively small is borne out by the fitting data below.

We validated these predictions by fitting the fALFF values of all bundles to the full forms of Eq. (3) and Eq. (5):

$$\text{Model}\;1:\;\text{fALFF}=\gamma_{1}\cdot CBV^{2}+\gamma_{2}\cdot OEF^{2}+e_{1}$$

$$\text{Model}\;2:\;\text{fALFF}=\gamma_{3}\cdot MTT^{2}+\gamma_{4}\cdot CMRO_{2}^{2}+e_{2}$$

All variables were z-scored before fitting. We found that both $\gamma_{1}$ and $\gamma_{2}$ terms were significant ($p_{\gamma 1}<0.001$ and $p_{\gamma 2}=0.02$), contributing comparably to fALFF (74.9% vs 25.1%). By contrast, the second term in equation (5) only contributes to 8.5% ($p_{\gamma 3}<0.001$ and $p_{\gamma 4}=0.73$), indicating that it is negligible. These results provide theoretical support for the relationships between fALFF and hemodynamic/metabolic features.

### 4. Myelination modulates hemodynamic- and metabolism- fALFF relationships in WM.

The above model predicts that bundles with higher $CMRO_{2}$ will exhibit lower fALFF, but the empirical data fits differed between WM bundle types (association vs projection; Fig. 2j), indicating that BOLD fluctuations are more weakly dependent on hemodynamic factors in some bundles. Similar heterogeneity was observed for CBV,CBF, and OEF. This heterogeneity reflects variations in WM microstructure, metabolism and composition. Previous reports have shown that WM bundles with varying myelination levels have distinct energy budgets^17^, and differences in energy utilization and capacity are related to mitochondrial density and capillary area, respectively (see Fig. 8 in reference^26^). Thus, we predicted myelin content could account for the heterogeneous metabolic and hemodynamic effects on fALFF.

**Fig. 3:**
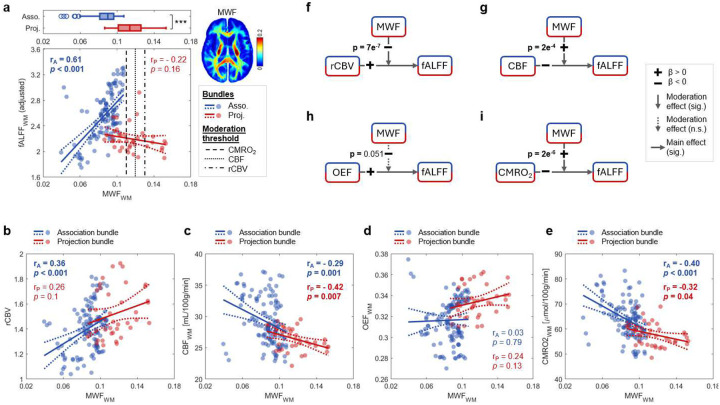
Myelination modulates the relationship between neurovascular metrics and WM BOLD fluctuations. **a**, Top: Boxplot of MWF in association and projection bundles (*** indicates p<0.001). Bottom: Scatter plot of fALFF versus MWF in association (blue) and projection (red) bundles, with vertical black lines indicating moderation thresholds of MWF for predictors rCBV (0.13, dash-dot line), CBF (0.12, dotted line) and ${\text{CMRO}}_{2}$ (0.11, dashed line). **b-e**, Scatter plots showing effects of MWF on rCBV (**b**), CBF (**c**), OEF (**d**) and ${\text{CMRO}}_{2}$ (**e**). **f**-**i**, Moderation effect of MWF on the relationship between rCBV (**f**), CBF (**g**), OEF (**h**) and ${\text{CMRO}}_{2}$ (**i**) with fALFF.

We first tested whether and how myelin content directly impacted fALFF, CBV, CBF, OEF and ${\text{CMRO}}_{2}$. We employed a published atlas of myelin water fraction (MWF)^27^, which represents the proportion of myelin-associated water within tissue relative to total tissue water), that was derived via multi-echo T2 relaxometry MRI. MWF was chosen as it has been histologically validated as a biomarker for myelin and shows significant variations between WM regions which may not be apparent with other techniques^28,29^. Specifically, we used the MWF atlas released by Dvorak *et al*.^27^ to derive bundle-wise MWF and quantified its relationship to fALFF in association and projection bundles. Association bundles, which had less myelin content than projection bundles (mean MWF=0.09±0.01vs.0.12±0.02,p<0.001; Fig. 3a, top), showed a significant positive correlation between fALFF and MWF (${\text{r}}_{\text{A}}=0.61,p=2{\text{e}}^{-9}$; Fig. 3a, bottom). In contrast, projection bundles exhibited a non-significant negative correlation (${\text{r}}_{\text{P}}=-0.22,p=0.16$). Meanwhile, MWF was significantly correlated positively with CBV (${\text{r}}_{\text{A}}=0.36,$ $p<0.001;{\text{r}}_{\text{P}}=0.26,p=0.10$; Fig. 3b), and negatively with CBF (${\text{r}}_{\text{A}}=-0.29,$ $p=0.001;{\text{r}}_{\text{P}}=-0.42$, p=0.007; Fig. 3c), and $CMRO_{2}$ (${\text{r}}_{\text{A}}=-0.40,p<0.001;{\text{r}}_{\text{P}}=-0.32,p=0.04$; Fig. 3e) in at least one bundle group, but no significant relationship was found with OEF (${\text{r}}_{\text{A}}=0.03,p=0.79;{\text{r}}_{\text{P}}=0.24$, p=0.13; Fig. 3d). These findings suggest that bundles with lower myelin content correspond to lower fALFF but higher baseline CBF and $CMRO_{2}$.

Next, we tested whether and how MWF moderates the relationship between fALFF and CBV (or CBF,OEF and $CMRO_{2}$) using the following canonical moderation model:

(7) $$\text{fALFF}=\beta_{0}+\beta_{1}\cdot CBV+\beta_{2}\cdot MWF+\beta_{3}\cdot CBV\cdot MWF+e$$

where CBV is the predictor, fALFF is the response variable, and MWF is considered a moderator. All variables were z-scored before fitting. We found that CBV had a positive and strong significant main effect on fALFF ($\beta_{1}=0.57,p<0.001$; Table 1) while MWF exhibited a negative but weaker main effect ($\beta_{2}=-0.18,p<0.01$). More importantly, the interaction term was negative and significant ($\beta_{3}=-0.32,p<0.001$), indicating that MWF moderates the CBV-fALFF relationship by weakening it. Specifically, in less myelinated bundles, CBV was a strong driver of fALFF, whereas in more heavily myelinated bundles, the CBV-fALFF relationship was dampened (Fig. 3f). The mathematical moderation threshold of MWF $\left(-\beta_{1}/\beta_{3}\right)$ for transiting CBV-fALFF relationship was approximately 0.13, above which all bundles are projection ones (Fig. 3a).

Similarly, we conducted additional moderation analyses using CBF,OEF and $CMRO_{2}$ as predictors (Table 1). Significant moderation effects of MWF were observed in the CBF-fALFF relationship ($\beta_{3}=0.27,p<0.001$; Fig. 3g) and the ${\text{CMRO}}_{2}$-fALFF relationship ($\beta_{3}=-0.32,p<0.001$; Fig. 3i), but not in the OEF-fALFF relationship (p=0.051; Fig. 3h). Notably, higher MWF attenuated the negative influence of OEF and $CMRO_{2}$ on fALFF, which is consistent with its dampening moderation on the CBV-fALFF relationship (Fig. 3a).

### 5. Modulation is achieved via influencing energy demand

**Fig. 4:**
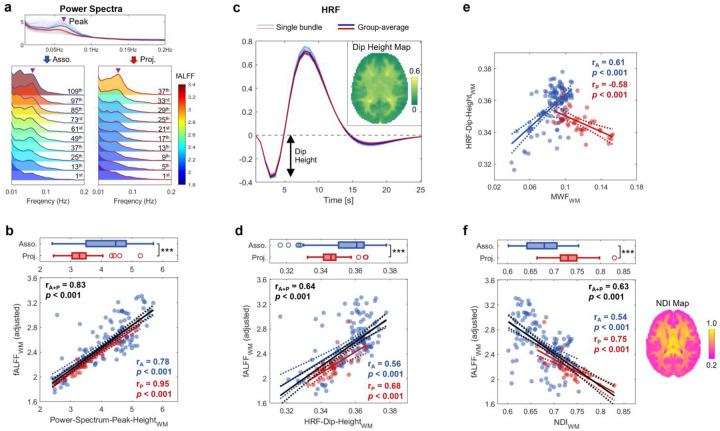
Relationship of WM fALFF with initial dip of HRF, the effect of myelination on the dip and effect of neurite density on fALFF. **a**, Top: Bundle-wise power spectra, with characteristic ‘peak’ location marked (purple arrow head). Bottom: Power spectra of ten representative association bundles (left ridgeline plot) and projection bundles (right), shaded by fALFF values. These bundles were selected uniformly from the fALFF-sorted bundle lists (ascending), with the 1^st^ bundle having the lowest and last bundle (i.e., 120^th^ for association or 40^th^ for projection) the highest. **b**, Top: Group comparison of peak height of power spectrum between association and projection bundles. Bottom: Correlation between fALFF and peak height of power spectrum across WM bundles (r = 0.83, p<0.001). **c**, Estimated bundle-wise HRFs, with an inset showing population-level map of dip height (upper right). **d**, Top: Group comparison of bundle-wise HRF dip height. Bottom: Correlation between fALFF and dip height of HRF across WM bundles (r = 0.63, p<0.001). **e**, Scatter plot of HRF dip height against MWF in association (blue) and projection (red) bundles. **f**, Top: Group comparison of neurite density index (NDI) between association and projection bundles. Bottom: Correlation between fALFF and NDI across WM bundles (r = −0.63, p<0.001), with a population-level map of NDI (right).

After deriving the relationships between myelin, hemodynamic parameters and fALFF by integrating atlases, we performed additional analyses of resting state power spectra to further demonstrate the effects of of myelination on BOLD signals. For each WM bundle and BOLD time course we calculated the power spectrum of the resting state fluctuations. Within each WM power spectrum, we identified a characteristic ‘peak’ at approximately 0.055Hz (marked by purple arrow heads in Fig. 4a), which was reported consistently in previous studies^16,30^. We found a strong correlation between the bundle-wise fALFF and the amplitude of this peak, regardless of bundle type (${\text{r}}_{\text{A}+\text{P}}=0.83,p<0.001$; Fig. 4b). Our previous studies demonstrated that this peak’s amplitude is proportional to the depth of the initial dip of the WM HRF^16^ and that this initial dip, particularly in deeper WM, increases as neurite density decreases, a behavior different to GM^30^. In line with these findings, we hypothesized that WM fALFF varies in parallel with the initial HRF dip, and so MWF will also influence this dip and WM fALFF will also increase as neurite density decreases. The HRF dip is a hemodynamic feature that reflects a transient increase in [dHb] due to increased local energy demands, and so relating it to fALFF links local metabolic changes with subsequent BOLD changes, while relating fALFF to neurite density links metabolic changes with changes in tissue microstructure and cellular composition.

To test this hypothesis, we evaluated the bundle-wise resting-state HRF (Fig. 4c) using voxel-wise HRF estimations from 137 healthy young subjects in Schilling *et al*.’s study^30^ (see Methods for more details). Consistent with our hypothesis, WM fALFF increases with a larger HRF dip, a trend observed across both association and projection bundles (${\text{r}}_{\text{A}+\text{P}}=0.64,p<0.001$; Fig. 4d, bottom). Moreover, Fig. 4e illustrates the relationships between MWF and the HRF dip in two types of bundles, suggesting that less myelinated fibers tend to exhibit smaller HRF dips in association bundles but larger HRF dips in projection bundles. This pattern mirrors the fALFF-MWF relationships (Fig. 3a), further supporting the role of myelination in modulating hemodynamic responses across WM bundles. Fig. 4f shows the relationship between fALFF and the neurite density index (NDI) obtained from the Neurite Orientation Dispersion and Density Imaging (NODDI) model analysis based on diffusion MRI data of the same subjects in Schilling *et al*.’s study^30^. fALFF decreases as the neurite density increases, implying that fALFF increases are driven by reductions in neurites and their replacement by other tissue components.

**Fig. 5:**
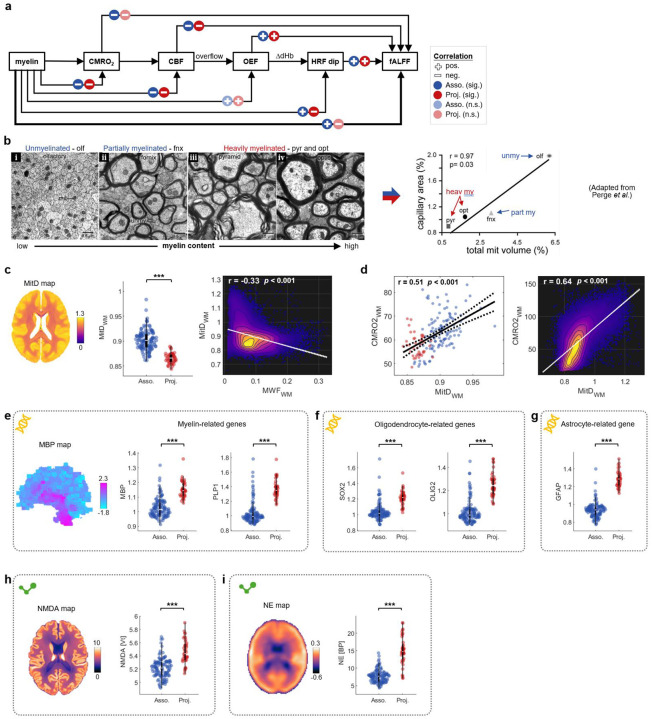
Summary of relatiohsips among myelination, metabolism, hemodemics, and fALFF, supported by histoglocial, cellular, neurotransmitter and gene-level evidence. **a**, Flow diagram summarizing observed relationships among myelin content, ${\text{CMRO}}_{2}$, CBF, OEF, HRF dip and fALFF. **b**, Left: Microscopy images of the cross-sections of four types of fibers in guinea pigs: unmyelinated olfactory nerve, partially myelinated fornix, heavily myelinated pyramid and optic nerve, with mitochondria indicated by solid gray balls. Right: Correlation between capillary area and mitochondrial volume across the four fibers, suggesting strong correlation between energy capacity and energy use (adapted from Perge *et al*.^26^). **c**, Whole-brain map of mitochondrial density (MitD) (left), group comparison between association and projection bundles for MitD (middle; *** p<0.001), and correlation between MitD and MWF across all WM voxels (right). **d**, Correlation between ${\text{CMRO}}_{2}$ and MitD across WM bundles (left) and across all WM voxels (right). **e-i**, Population-level maps and group comparisons between association and projection bundles for myeline-related genes (MBP and PLP1) (**e**), oligodendrocyte-related genes (SOX2 and OLIG2) (**f**), astrocyte-related gene (GFAP) (**g**), PET-measured NMDA receptor binding (**h**), and PET-measured norepinephrine transporter binding (**i**).

Combining our findings, less myelinated fibers at rest show higher ${\text{CMRO}}_{2}$ (Fig. 3e), higher CBF (Fig. 3c), a smaller HRF dip (Fig. 4d) and lower fALFF (Fig. 2b), following a sequential causal relationship (Fig. 5a). This link between myelination, metabolism, and neurovascular couplings is consistent with evidence from previous histological studies. For example, Perge *et al*.^26,31^ showed that less myelinated fibers (e.g., unmyelinated olfactory axons) tend to have higher mitochondrial concentrations and greater capillary areas (Fig. 5b), corresponding to higher tissue oxygen consumption and greater oxygen (and blood) supply capacity. By contrast, heavily myelinated fibers, representing projection fibers (Fig. 3a), have lower mitochondria concentrations and capillary areas (Fig. 5b), as well as lower ${\text{CMRO}}_{2}$ and CBF (Fig. 2f), suggesting that they may not rely as much on delivery of oxygen.

To substantiate the relationships of WM BOLD and composition to cellular metabolism, we combined our data with information on mitochondrial density provided by an atlas produced by Mosharov *et al*.^32^ (see Methods). By performing bundle-wise and voxel-wise analyses of human brain mitochondrial densities we confirmed that the association bundles, which are less myelinated, possess higher mitochondrial density than projection bundles (p<0.001; Fig. 5c) and mitochondrial density was inversely correlated with MWF across all WM voxels (r = −0.33, p<0.001). Moreover, ${\text{CMRO}}_{2}$ positively correlated with mitochondrial density at both bundle and voxel levels (Fig. 5d).

We expect that the heterogeneity of WM composition, metabolism and BOLD effects are the result of synthetic and physiologic processes that are controlled by genes. To explore this proposition, we combined our imaging structural data with brain-wide gene expression maps from the Allen Human Brain Atlas^33^, and neurotransmitter concentrations using publicly available PET atlases^34,35^. We thereby assessed bundle-wise gene expression and neurochemical levels (see Methods). Compared to association bundles, projection bundles exhibited higher expression levels of myelin basic protein (MBP), proteolipid protein 1 (PLP1) (Fig. 5e), as well as SRY-box transcription factor 2 (SOX2), oligodendrocyte transcription factor 2 (OLIG2) (Fig. 5f), and glial fibrillary acidic protein (GFAP) (Fig. 5g), suggesting a greater presence of myelin, oligodendrocytes and astrocytes within projection bundle volumes. Meanwhile, projection bundles showed higher levels of N-methyl-D-aspartate (NMDA) receptor density (Fig. 5h) and higher norepinephrine (NE) transporters (Fig. 5i), whose presence may be relevant for the interpretation of specific metabolic pathways as discussed below.

### 6. Myelination and axon diameter

**Fig. 6:**
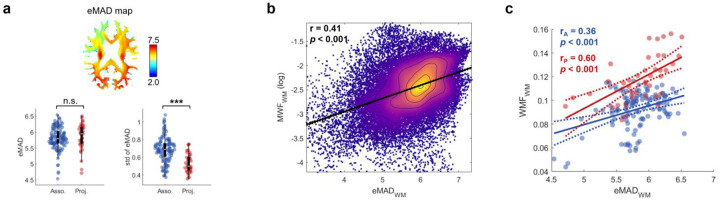
Axon diamater and its relathion with myelination. **a,** Top: Population-map of eMAD. Bottom: Group comparison of eMAD and its standard deviation between association bundles (blue) and projection bundles (red). **b,** Scatter plot showing the correlation between MWF (log scale) and eMAD across whole-brain WM voxles. **c,** Scatter plot showing correlations between MWF and eMAD wihtin association bundles (blue) and projection bundles (red).

Axons in WM vary in diameter as well as in myelin content so we also examined the dependence of MWF on axon diameter using an atlas of estimated mean axon diameter (eMAD) derived from diffusion MRI^36^. Although the bundle-wise eMAD did not differ significantly between association and projection bundles (p=0.34), the within-bundle variability of eMAD was significantly greater in association bundles compared to projection bundles (p<0.001) (Fig. 6a). Significant correlations between eMAD and MWF were observed at both voxel and bundle levels (Fig. 6b,c). However, no significant relationship was observed between bundle-wise eMAD and fALFF in either the association or projection groups (**Fig. S4**). Thus axonal size per se does not appear to be a main driver of BOLD signal fluctuations at rest.

### 7. Myelination affects BOLD signal change in WM during visual task

**Fig. 7:**
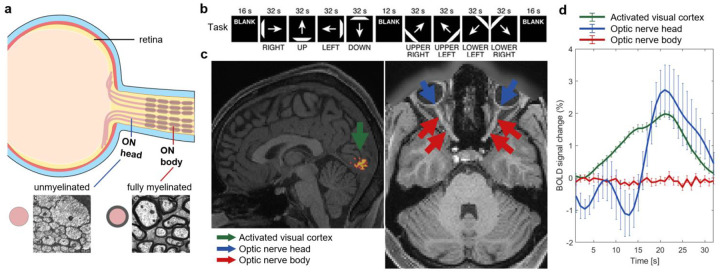
BOLD signal changes in unmyelinated and myelinated portion of the optic nerve during visual stimulation. **a,** Schematic diagram of optic nerve (ON) anatomy, including the unmyelinated ON head and the fully myelinated ON body (micrographs adapted from Perge *et al*.). **b**, Visual task paradigm involving moving bars across the visual field (adapted from Benson *et al*.^38^). **c**, Regions of interest shown on one subject’s anatomical image: task-activated visual cortex (green arrow), optic nerve head (blue arrow) and optic nerve body (red arrow). **d**, BOLD signal changes measured in the activated visual cortex (green), unmyelinated optic nerve head (blue) and myelinated optic nerve (red).

A convincing demonstration of the effects of myelination on BOLD signals is provided by considering the human optic nerve, which is unmyelinated near the eye orbit (referred to as the “optic nerve head”) but becomes heavily myelinated after several millimeters (optic nerve body) (Fig. 7a)^31,37^. To evaluate the spatial dependence of BOLD responses within the same nerve we analyzed high resolution HCP 7T retinotopy datasets^38^ (spatial resolution = 1.6mm^3^) from 20 healthy adults who underwent a visual stimulation task involving light bars moving across the visual field during fMRI image acquisitions (Fig. 7b). BOLD signal changes evoked by the stimulus were calculated separately in the GM of the visual cortex (to verify task activation), as well as the optic nerve head and optic nerve body. A strong, time locked BOLD response was observed in the unmyelinated optic nerve head (Fig. 7c), as well as the visual cortex. In contrast, no significant BOLD signals were observed in the fully myelinated optic nerve body just a few millimeters downstream. Previous studies have shown this area of more complete myelination has a lower density of mitochondria^31,37^.

## Discussion

Functional MRI relies on the ability to relate BOLD signals, and their changes during a task or over time, to corresponding changes in neural activity. In practice, in GM, larger or more widespread BOLD signals are interpreted as indicating greater underlying neural engagement, but the vaidity of this inference assumes a direct connection between MRI signal decay rates and the electrical activity (spiking rates or local field potentials) of populations of neurons within a voxel, and an understanding of what biophysical factors and physiological mechanisms may affect those relationships MRI signals. BOLD signals in WM have been overlooked partly because of a lack of knowledge of what factors and processes in tissues explain their origins and characteristics. This study aims to address this lack and in so doing makes four major contributions to understanding and interpreting BOLD signals in WM. First, we established the associations of WM BOLD fluctuations (quantified by fALFF) with hemodynamic and metabolic properties represented by CBV, CBF, OEF and ${\text{CMRO}}_{2}$ during a resting state, using empirical data from multiple sources as well as by theory. Second, we demonstrated that myelin content and neurite density influence fALFF and the HRF and modulate their relationships with these hemodynamic and metabolic features, findings that were further supported by converging biological, genomic and neurotransmitter evidence. Third, we observed that BOLD activation in response to a stimulus was significant in unmyelinated regions of nerve fibers, but there is minimal activation in fully myelinated fibers, suggesting a myelin-dependent gating of BOLD responses in WM. Fourth, collectively these data demonstrate how BOLD effects in WM are heterogeneous and point to the need for differentiating types of fibers (e.g. association vs projection) in further analyses of functional imaging data from WM.

### Relationship of fALFF with hemodynamic and metabolic properties

To quantify fALFF in WM, where BOLD contrast to noise is relatively low, we adopted an unbiased sampling approach within each WM bundle (Figs. 2a and **S1**), balancing the reliability and feasibility of bundle-wise fALFF estimation. Resting-state fALFF within WM, although generally lower compared to the connected GM (**Fig. S5**), exhibited substantial spatial heterogeneity (Fig. 2b), but was significantly associated with hemodynamic (i.e., CBV and CBF) and metabolic (i.e., OEF and ${\text{CMRO}}_{2}$) variations (Fig. 2g–j). The positive fALFF-rCBV relationship in WM (Fig. 2g) suggests that a substantial portion of spatial variation in WM fALFF can be explained by regional differences in baseline CBV, consistent with prior research in GM, where fALFF was positively correlated with hyperoxia-BOLD signal changes (a proxy for venous CBV) in healthy adults^39^. The negative correlations of fALFF with both CBF and ${\text{CMRO}}_{2}$ are in line with the known CBF-${\text{CMRO}}_{2}$ coupling in GM^40,41^. These patterns support a physiological model of WM at rest, wherein higher oxygen consumption is more than satisfied by higher blood flow resulting in lower oxygen extraction fractions, lower [dHb] levels, and ultimately smaller fractional BOLD variances (Fig. 4f). While the precise explanation of overflow is not yet fully understood, it is thought to serve as a means of delivering sufficient oxygen to match the aerobic needs of glucose metabolism, independent of any oxygen-sparing requirements of anaerobic processes^1^. Indeed, the increase in flow may not be driven by oxygen demands alone. Another observation to emphasize is that fALFF and absolute changes in $R_{2}^{*}$ vary in different ways with ${\text{CMRO}}_{2}$. Higher values of ${\text{CMRO}}_{2}$ may induce greater changes in $R_{2}^{*}$, but lower fractional variations measured by fALFF. The net contribution to $\delta R_{2}^{*}$ is, from Equation 4, a competition between increases in ${\text{CMRO}}_{2}$ and decreases in MTT. Notably, in this study, we employed simple linear models for all regression analyses, which may not provide the best fit for characterizing the relationships between variables of interest. However, our primary goal was to emphasize only the existence and directionality of these relationships, but more accurate fitting, particularly at the voxel-wise level, may be warranted as more data becomes available.

### Myelin and energy metabolism

Myelin plays a significant role in modulating WM BOLD fluctuations via its influence on metabolism (Fig. 3). The differential effects of myelin content on CBF, ${\text{CMRO}}_{2}$ and fALFF for association and projection fibers highlight the varying nature of this role. Specifically, as MWF increased, both ${\text{CMRO}}_{2}$ and CBF exhibited a steep decline in less myelinated (association) bundles, whereas there is a more gradual decrease in highly myelinated (projection) bundles (Fig. 3c, e), indicating increases in myelination have a larger effect in association fibers. Additionally, the sensitivity of fALFF to changes in ${\text{CMRO}}_{2}$ and CBF was also higher in association bundles (Fig. 2h, j), collectively supporting the observation that the rate of fALFF change with respect to MWF differs between bundle groups (Fig. 3a). These findings converge with our moderation results, which show that myelin attenuates the dependence of fALFF on hemodynamic factors and energy use—acting as a potential ‘buffer’ (Fig. 3g, i).

### Myelin and astrocytes/NE/NMDA

In light of established findings, we cannot fully exclude the contribution of anaerobic metabolism in WM in a resting state. For example, a recent study of WM cells showed that “unlike GM astrocytes, which interact at synapses, WM astrocytes interact with neurons at the nodes of Ranvier, oligodendrocytes and vasculature, supporting network activity and myelination”^42^. In addition, the ‘lactate shuttle’ mechanism^43^ may reduce oxygen demand by supplying lactate as an alternative energy source^44,45^, with NE stimulating glycogenolysis in astrocytes^46^ and NMDA receptor signaling at the “axon-oligo synapse”^47^. Stimulated by these observations, we integrated imaging data with gene expression and neurotransmitter maps and our data confirm that projection bundles exhibit higher levels of astrocytes, NE and NMDA (Fig. 4j–l), alongside lower ${\text{CMRO}}_{2}$ (Fig. 2f), suggesting a possible role for anaerobic metabolism in shaping the observed metabolic pattern in WM. Overall, these findings demonstrate that neurovascular coupling, energy demands and supply mechanisms are not uniform within WM.

### Myelin and axon diameter

From Fig. 6a it is apparent that projection bundles have comparable axon diameters but higher myelin content than association bundles (Fig. 2b), suggesting either denser axonal packing and/or thicker myelin sheaths in projection bundles, consistent with supporting histological evidence (Fig. 4g). Additionally, the larger variability of axon diameters within association bundles is also in line with histological evidence.

### BOLD activation in optic nerve

Ganglion cell axons are unmyelinated within the retina but fully myelinated within the optic nerve, even though axon caliber is nearly identical in both regions^31^. The observed differences in BOLD signal changes in the optic nerve in response to visual stimuli are therefore attributable to differences in myelination and mitochondrial density rather than axon diameter. Furthermore, the negligible BOLD responses measured at 7T in the fully myelinated optic nerve body (Fig. 7d) are consistent with the observation of lower fALFF measured at 3T in myelinated projection bundles in (Fig. 2b), reinforcing the modulatory role of myelin.

### Limitations

Beyond the limitations highlighted above, this study has two additional caveats. First, CBF, OEF and ${\text{CMRO}}_{2}$ were derived from the same dataset, and therefore may not represent fully independent measures. Second, all analyses were conducted at a population level, using atlases, so that some errors may arise from imperfect co-registration across individuals and datasets. Future studies leveraging subject-specific datasets are anticipated to enhance the precision of these comparisons.

### Implications and impact

In summary, this study offers new insights into the relationships between functional, structural and biophysical properties of WM to better understand the factors that drive BOLD effects both in a resting state and in response to a stimulus, providing an improved understanding of how to interpret BOLD signals in WM. It demonstrates the relationships of BOLD metrics to hemodynamic changes and the modulatory role of myelin and tissue microstructure in influencing BOLD signals, and it reveals the heterogeneous nature of BOLD effects in different types of WM bundle.

## Materials and methods

### Definition of GM-WM-GM unit, association and projection bundles

We defined a GM-WM-GM unit as a structural triplet consisting of two GM parcels and a WM bundle connecting them (Fig. 1B). Specifically, we extracted over 260,000 association and projection streamlines from the HCP1065 population-averaged tractography atlas^21^ and reassigned each streamline to a specific WM bundle according to its connected GM pair. The GM parcels included the parcels predefined by the Schaefer 100 atlas^22^, cerebellar cortex and cervical portion of spinal cord (a pseudo-GM parcel). Details on streamline reassignment are provided in the supplementary methods. To ensure sufficient sampling for further analysis, bundles with fewer than 500 voxels (= 4,000mm^3^) were excluded. This resulted in 120 GM-WM-GM units with association bundles and 40 units with projection bundles.

### Evaluation of bundle-wise fALFF at rest

Resting state fMRI data used for fALFF evaluations were sourced from the HCP Young Adult dataset^48^, comprising 120 randomly selected subjects (60 females, aged 26–35). The fMRI data were acquired using a multiband gradient-echo EPI sequence (TR/TE = 720/33.1 ms, voxel size = 2 mm isotropic, and 1200 time points) and were minimally preprocessed by the HCP pipeline^49^. Additional preprocessing included regressing out head motion, cardiac and respiratory signals, as well as linear detrending. To mitigate possible volume effects on bundle-wise fALFF, we randomly sampled the same number of voxels (n = 500) from each bundle and averaged their time series. The power spectrum of each bundle’s averaged time series was evaluated using the Welch method^50^ and the fALFF value was computed as the ratio of the average spectral amplitude within the 0.01–0.1 Hz band to the average amplitude across the full frequency range above 0.01 Hz. This procedure was repeated 50 times and the resulting 50 fALFF values for each bundle were averaged.

### Evaluation of bundle-wise rCBV, CBF, OEF and ${\text{CMRO}}_{2}$ at rest

Bundle-wise rCBV values were derived from a population-level rCBV atlas (https://github.com/eliesfuster/cbv_atlas) developed by Fuster-Garcia *et al*.^51^. This atlas was constructed from rCBV maps of 134 glioblastoma patients (mean age = 60 years) estimated based on dynamic susceptibility contrast T2* perfusion imaging data, with tumor-affected regions excluded. The bundle-wise rCBV value was determined by averaging rCBV values across all voxels within each bundle. Similarly, CBF, $\text{CMR}{\text{O}}_{2}$ and OEF values for each bundle were calculated by averaging their respective values across bundle voxels from group-averaged maps (https://owncloud.cubric.cf.ac.uk/s/UtdmuMqU80rVRY7) generated by Driver *et al*.^25^, based on breath-hold calibrated fMRI in 33 healthy subjects (mean age = 25 years).

### Evaluation of bundle-wise myelin water fraction (MWF) and estimated mean axon diameter (eMAD)

Bundle-wise MWF and eMAD values were estimated based on population-level atlases published by Dvorak *et al*.^52^ and Gast *et al*.^36^, respectively. The MWF atlas was constructed using multi-echo T2 imaging data (3D GRASE with TR/TE = 1073/8ms, ΔTE=8ms, 48 echoes) from 100 healthy subjects (median age = 38 years). The eMAD atlas (https://github.com/HilaGast/AxSI.git) was generated using axonal spectrum imaging applied to multi-shell diffusion MRI data (TR/TE = 5520/89.5 ms, Δ/δ=43.1/10.6ms, voxel size = 1.25 mm isotropic, b-values = 1000/ 2000/3000 s/mm^2^) from 324 random subjects (180 females, mean age = 28.9 years) in the HCP Young Adult database. Bundle-wise values were evaluated by averaging voxel-wise measurements within each bundle.

### Estimation of bundle-wise HRF feature and NDI

Bundle-wise HRF features were estimated using HRF data from our previous study^30^. Briefly, resting state fMRI data from 137 random subjects (72 females, aged 22–35) from the HCP Young Adult dataset were analyzed. Voxel-wise HRFs were estimated from resting-state time series using a blind deconvolution approach^53^, implemented via the rsHRF toolbox^54^. This method does not require prior assumptions about the HRF shape and is based on the notion that prominent BOLD signal peaks correspond to major spontaneous neural events. For each subject, the initial dip height of the HRF was extracted from each voxel in MNI space, yielding a subject-specific parametric map. A population-level HRF dip map was then generated by averaging across all subjects (see reference^30^ for more methodological details). Bundle-wise HRF dip height was evaluated by averaging the dip values of all voxels within each bundle on the population-level map.

### Evaluation of bundle-wise mitochondrial density (MitD)

Bundle-wise MitD values were estimated using a whole-brain MitD map (http://humanmitobrainmap.bcblab.com) released by Mosharov *et al*.^32^. MitD was quantified using a combination of citrate synthase activity and mitochondrial DNA density^32^. The map was generated from enzyme assays and snRNA-seq data obtained from a brain tissue slab of a 54-year male donor, and extrapolated to the whole-brain via backward linear regression informed by multimodal imaging data (see reference^32^ for details). Bundle-wise MitD was computed by averaging voxel-wise MitD values within each WM bundle.

### Evaluation of bundle-wise gene expression level

Bundle-wise gene expression levels were estimated using brain-wide gene expression maps (specifically MBP, PLP1, SOX2, OLIG2 and GFAP genes in this study) from the Allen Human Brain Atlas (https://neurosynth.org/genes). These maps were derived from microarray analyses of brain samples from six healthy donors (aged 18–68). In brief, isolated RNA was used for microarray profiling of ~900 samples per brain, followed by normalization and coregistration to native MRI space via histological micrographs as an intermediate step. The resulting maps from all donors were transformed into MNI space and averaged to produce a population-level gene expression map (see reference^33^ for more methodological details). Bundle-wise gene expression level was then computed by averaging voxel values within each bundle on the population-level map.

### Evaluation of bundle-wise neurotransmitter receptors and transporters

Bundle-wise NMDA receptor density and NE transporters were estimated from population-level PET maps (https://netneurolab.github.io/neuromaps). The NMDA receptor density map was generated using PET data from 29 healthy subjects (mean age = 40.9±12.7 years) with the radioligand [^18^F]GE-179, which binds within the ion channel of open, activated NMDA receptors^34^. The total volume of distribution (V_T_) of [^18^F]GE-179 was used to quantify the receptor density. The NE map was created using PET data collected from 10 healthy subjects (mean age = 33.3 years)^35^ with the radioligand S, S-[^11^C]O-methylreboxetine (MRB). Voxel values represent the binding potential of radioligand. For both NMDA and NE maps, bundle-wise values were obtained by averaging voxel values within each bundle on the respective population-level maps.

### Visual task fMRI analyses for optic nerve

We analyzed HCP 7T retinotopy datasets^38^ acquired from 20 healthy adults (mean age = 28 years). For each subject, we used the last two runs (RETBAR1 and RETBAR2; TR/TE = 1000/22.2 ms, voxel size = 1.6 mm isotropic, 300 time points), during which high-contrast, dynamically textured bars slowly swept across the visual field in eight directions (RIGHT, UP, LEFT, DOWN, UPPER-RIGHT, UPPER-LEFT, LOWER-LEFT, LOWER-RIGHT; see Fig. 7b for the eight stimulus blocks). Each block lasted 32s, including a 4s blank period at the end. Each run also included 16-s blanks at the beginning and end, and a 12-s blank in the middle, serving as rest periods.

For each subject, we identified the task-activation region in visual cortex based on the minimally preprocessed dataset and manually segmented areas of optic nerve and optic nerve head on T1 images which were coregistered to the subject’s fMRI space. Voxel-wise time series within each region were averaged to obtain regional time-courses, denoted as S(t). Percent BOLD signal change was calculated as:

$$\text{BOLD}\;\text{signal}\;\text{change}\;(\%)=\frac{S(t)-S_{\text{rest}}}{S_{\text{rest}}}\times 100\%$$

Signal changes were then averaged across all eight stimulus blocks and across all subjects, separately for the activated cortex, optic nerve and optic nerve head.

## Figures and Tables

**Table 1.** Moderation effect of MWF on relationship between each predictor and fALFF (z-scored variables).

Predictor	Main effect of predictor, $\beta_{1}$	Main effect of MWF, $\beta_{2}$	Moderation effect of MWF, $\beta_{3}$	Moderation threshold of MWF
rCBV	0.57 (*p* = 1e^−13^) ***	−0.18 (*p* = 0.009) **	−0.32 (*p* = 7e^−7^) ***	0.129
CBF	−0.38 (*p* = 8e^−6^) ***	−0.08 (*p* = 0.32)	0.27 (*p* = 2e^−4^) ***	0.122
OEF	0.39 (*p* = 1e^−6^) ***	−0.01 (*p* = 0.90)	−0.14 (*p* = 0.051)	-
CMRO_2_	−0.26 (*p* = 0.002) **	−0.08 (*p* = 0.34)	0.35 (*p* = 2e^−6^) ***	0.109

Note: $\beta_{1},\beta_{2}$, and $\beta_{3}$ were calculated based on z-scored predictor, z-scored MWF and z-scored fALFF.
